# Giant Protruding High-Grade Undifferentiated Pleomorphic Sarcoma Arising in a Keloid Scar on the Abdominal Wall

**DOI:** 10.1155/2020/4898965

**Published:** 2020-07-24

**Authors:** Hideyuki Kinoshita, Toshinori Tsukanishi, Takeshi Ishii, Hiroto Kamoda, Yoko Hagiwara, Sumihisa Orita, Kazuhide Inage, Naoya Hirosawa, Seiji Ohtori, Tsukasa Yonemoto

**Affiliations:** ^1^Department of Orthopedic Surgery, Chiba Cancer Center, 666-2 Nitonacho, Chuo-ku, Chiba 260-8717, Japan; ^2^Department of Orthopaedic Surgery, Graduate School of Medicine, Chiba University, 1-8-1 Inohana, Chuo-ku, Chiba 260-8670, Japan

## Abstract

Undifferentiated pleomorphic sarcoma (UPS) is a high-grade, aggressive, soft tissue sarcoma that is often fatal. Although there are reports describing associations of sarcoma and skin lesions such as burns, radiation, and trauma, to our knowledge, UPS development in a keloid scar has not been reported. Herein, we present the case of a 76-year-old woman who had undergone surgery for endometrial cancer, 5 years before. She presented with a protruding lesion that was continuous to a keloid scar on the abdominal wall. The tumor appeared clinically malignant as it was protruding and doubled in size within three weeks, reaching approximately 6 × 6 × 2 cm. Since the tumor was diagnosed as UPS after pathological evaluation by needle biopsy, wide resection was performed. Intraoperatively, the tumor was apparently continuous to the keloid, protruding and pedunculated outside the body, and had not invaded the abdominal cavity. Histopathological examination of the resected tumor showed evidence of UPS and no suspicion of metastasis of endometrial cancer. No recurrence, metastases, or other complications were noted 6 months after surgery. The current case study reminds us that keloids may cause high-grade sarcoma such as UPS, and careful follow-up is required.

## 1. Introduction

Undifferentiated pleomorphic sarcoma (UPS) is a high-grade, aggressive, soft tissue sarcoma, and the preferred name for the sarcoma is, previously called, malignant fibrous histiocytoma (MFH) [1]. Since UPS has a high rate of lung metastasis and is sometimes fatal, early diagnosis is imperative. There are many reports describing associations between sarcomas, such as osteosarcoma and UPS and skin lesions, such as burns, radiation, and trauma [2]. Keloid is a benign fibroproliferative disorder of the skin that presents in scar tissue when a wound heals. Although keloid scars can be difficult to be differentiated from dermatofibrosarcoma protuberans (DFSP) [3], there are no reports of UPS development in a keloid scar.

Here, we present an unusual case of high-grade undifferentiated pleomorphic sarcoma arising in a keloid scar.

## 2. Case Presentation

**Figure 1:**
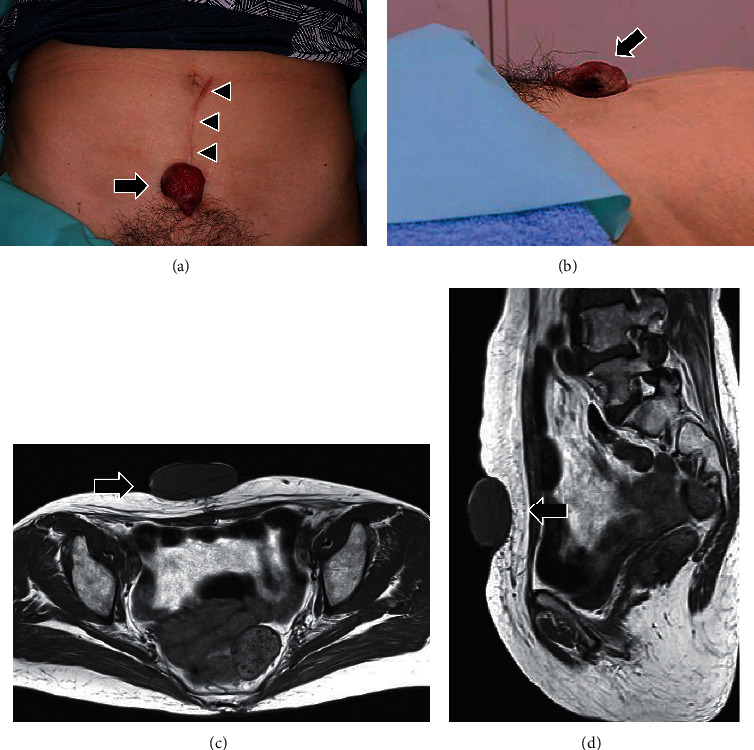
Frontal (a) and lateral (b) view of the large protruding tumor (arrow) located on the abdominal wall. The lesion measured 3 × 3 × 2 cm at the first visit. A keloid scar (arrowhead) is visible on the abdominal wall due to partial resection of endometrial cancer. Axial (c) and sagittal (d) view of magnetic resonance imaging revealing a tumor protruding subcutaneously with no continuity to the abdominal cavity (arrow).

**Figure 2:**
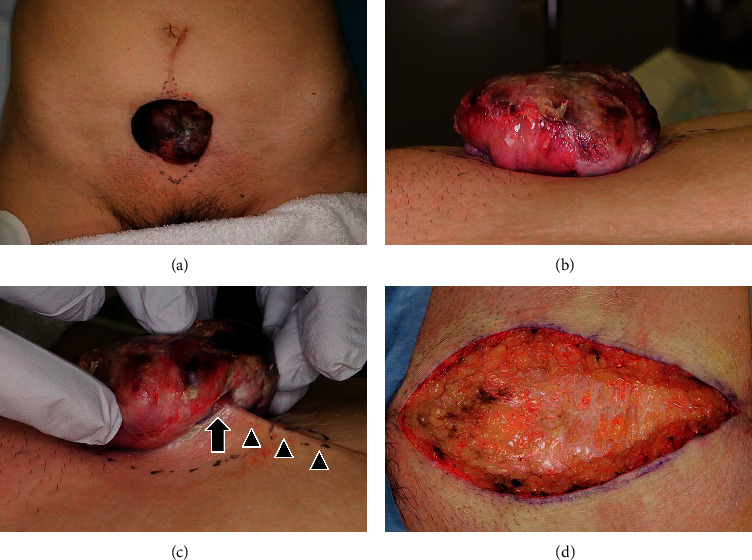
Frontal (a) and lateral (b) view of the tumor after three weeks. The tumor has rapidly increased in size to 6 × 6 × 2 cm and protruded further. (c) The tumor is observed to be continuous with the keloid scar (arrowhead), protruding outside the body, and pedunculated (arrow). (d) Intraoperatively, the tumor had not invaded the abdominal cavity.

**Figure 3:**
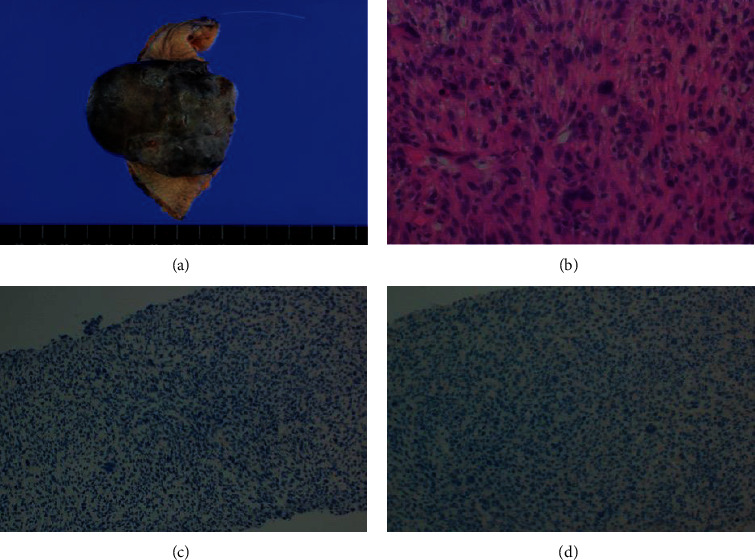
(a) Gross view of the resected tumor. The tumor is observed to be continuous to the keloid and measures approximately 6.2 × 6 × 3.2 cm. (b) Histopathological examination following hematoxylin and eosin (HE) staining revealing short pleomorphic spindle cells and polygonal cells with a storiform pattern, accompanied by abundant mitotic activity, suggesting undifferentiated pleomorphic sarcoma. The tumor is negative for cytokeratin AE1/AE3 (c) and CAM5.2 (d) on immunohistochemical staining.

A 76-year-old woman with a 4-month history of a tumor protruding from the abdominal wall presented to our orthopedic outpatient department. She had a medical history of partial resection for endometrial cancer 5 years before. Although the skin developed keloid scars after the resection, the clinical course was good with no recurrence or wound infection. However, 4 months prior to presentation, a tumor emerged from the keloid scar on the abdominal wall and protruded outside the skin, growing rapidly. On physical examination, a protruding lesion continuous with the keloid scar was noted on the abdominal wall, measuring approximately 3 × 3 × 2 cm (Figures 1(a)-1(b)). The needle biopsy specimen was characterized by high cellularity and marked nuclear pleomorphism and accompanied by abundant mitotic activity and a spindle cell morphology, suggesting pleomorphic and spindle cell sarcoma. Furthermore, magnetic resonance imaging (MRI) 1 week later revealed a tumor measuring 5 × 4 × 3 cm protruding subcutaneously with no continuity to the abdominal cavity (Figures 1(c)-1(d)). The tumor rapidly increased in size to 6 × 6 × 2 cm and protruded further in the following two weeks (Figures 2(a)-2(b)). Wide resection of the tumor including the keloid scar and fat layer immediately above the rectus sheath was performed. Intraoperatively, the tumor was apparently continuous to the keloid, protruding outside the body, and pedunculated (Figure 2(c)). Furthermore, we observed that the tumor had not invaded the abdominal cavity (Figure 2(d)). The resected tumor was continuous to the keloid, measuring approximately 6.2 × 6 × 3.2 cm (Figure 3(a)). Histopathological examination following hematoxylin and eosin staining revealed short pleomorphic spindle cells and polygonal cells with a storiform pattern, accompanied by abundant mitotic activity, similar to the histology obtained on needle biopsy (Figure 3(b)). Moreover, the tumor was negative for smooth muscle actin, h-caldesmon, cytokeratin AE1/AE3, CAM5.2, and CD34 (Figures 3(c)-3(d)), and there was no suspicion of metastasis of the endometrial cancer, suggesting a diagnosis of high-grade UPS. The postoperative period was uneventful. In the 6 months of follow-up, the patient did not experience any recurrence, metastasis, novel keloid scars, or other complications.

## 3. Discussion

The UPS tumor category, previously known as pleomorphic MFH, is a heterogeneous group of disorders. It is the fourth most common soft tissue sarcoma and has a slight male preponderance. It is mainly composed of mesenchymal lineages that belong to established sarcoma subgroups such as liposarcoma, leiomyosarcoma, and fibrosarcoma [1]. UPS is common in the deep soft tissue of the extremities and rarely occurs on the body surface of the skin with a stalk. The etiology of UPS remains unknown. Histologically, UPS has pleomorphic spindle cells with abundant mitotic figures, associated regions of necrosis, and hemorrhage, while UPS is negative for S-100, CD34, and cytokeratin on immunostaining. Although wide surgical excision is the best procedure for UPS, postoperative multidisciplinary treatment with chemotherapy and radiation therapy is often required, depending on the tumor size, grade, and stage.

There are many reports on sarcoma and skin lesions such as burns, radiation, and trauma. An 80-year review of the literature on burn cases showed 412 case reports of neoplasm arising in burn scars [2]. Furthermore, Kowal-Vern and Criswell reported that, among neoplasms arising in burn scars, although squamous cell carcinoma (SCC) was the most common at 71% cases, 5% of them were sarcomas [4]. Radiation can also induce sarcomas although this is rare. In most cases, radiotherapy for cancer treatment leads to radiation-induced sarcoma (RIS). Although radiation-induced malignancies may cause any subtypes of sarcoma, osteosarcoma and UPS are the most common among them. Radiation may also develop other malignancies, including leukemia, lymphoma, and solid tumors from other germ layers [5]. Significant trauma has been also reported to be a rare risk factor for the development of neoplasia. Witwicki et al. reported that sarcoma arose at a fracture site after internal fixation and open reduction [6].

The pathogenesis of malignancies accompanied with skin lesions such as burns and trauma is not precisely understood. Initially, it was hypothesized that the tissue damaged by burn and trauma released toxins, inducing cellular mutations [7]. Another theory regarding the occurrence of skin malignancy after trauma is the displacement of live epithelial cells into the deep tissue with a concurrent inability of the tissue damaged by trauma to regulate for the invading cells [8]. Furthermore, Nakanishi et al. reported that SCCs, which commonly harbor p53 mutations, are the commonest malignancy arising in burn scars [9]. In contrast, the etiology of RIS has been elucidated. The increase in ionizing radiation induces an increase in DNA breaking, eventually leading to the replication of aberrant cells. Sarcomas arisen from the mesenchymal origin indicates one of the highest proportions of radiation-induced cancers, probably because of a massive amount of active dividing tissue over a large surface area. [10].

Keloids are fibroproliferative disorders of the skin that result from abnormal healing of injured or irritated skin [11]. Although keloids are thought to be derived from epigenetic alterations in fibroblast DNA and acetylation of histone proteins, keloid scar formation and progression is complex and poorly understood. Differential diagnoses of keloids include complications of acne, hyperplastic scars, and keloidal dermatofibroma. DFSP can be misdiagnosed as a keloid scar [3].

To our knowledge, this is the first case to report UPS development in a keloid scar. Although our patient had a history of endometrial cancer, suggesting the possibility of recurrent disease, the present case had no continuity from the abdominal cavity on MRI. Furthermore, there was no pathological evidence of any recurrence or metastasis of endometrial cancer. In addition, as a clinical finding, a tumor continuous to the keloid was observed; therefore, the tumor was thought to have originated from the area of the keloid.

In conclusion, we reported a rare case of high-grade UPS arising in a keloid scar. Keloids may cause high-grade sarcomas such as UPS, and careful follow-up is required.
